# Supplementary material for: Effects of manufacturing modality, primer, and adhesive polymerization on the shear bond strength of customized lingual brackets to glazed zirconia: An in vitro study
Source: PLoS One. 2025 Dec 4;20(12):e0338181. doi: 10.1371/journal.pone.0338181 (PMC12677577; doi:10.1371/journal.pone.0338181)
Supplement: Attachment — Submitted filename: Response to reviewer R1.docx [file pone.0338181.s003.docx]

November 7^th^, 2025

Dear Editorial Board, PLOS ONE,

We have revised the manuscript thoroughly according to the comments of the reviewers. Any revisions made in our manuscript document were highlighted in red. Please help us review the manuscript again.

Sincerely,

---------------------------------

**Reviewer #1:**

***Comment:*** First page
- What is the difference between "Viet Nam" and "Vietnam". Uniform Journal style would be appreciated.
***Response:*** Thank you for your comment. We standardized all occurrences to **“Vietnam”** in affiliations and text for consistency with journal style; no instances of “Viet Nam” remain.

***Comment:*** Title
- Please italicize "in vitro".
- No capital letters, please.
***Response:*** Done. Updated title: “Effects of manufacturing modality, primer, and adhesive polymerization on the shear bond strength of customized lingual brackets to glazed zirconia: An *in vitro* study.” An is capitalized as per PLOS ONE policy.

***Comment:*** Abstract
- Please note that 300 are allowed for with this section, so maximize information. Remember that this section is most important, since future readers will switch to your full text after having read your Abstract.

***Response:*** Revised to a concise abstract ≤300 words, emphasizing objectives, design, materials, primary/secondary outcomes, statistics (including post-hoc), and key findings.

***Comment:*** - Please provide materials (Single Bond Universal and Z-Prime Plus) used.

***Response:*** Thank you for your comment. We have added brand names Single Bond Universal and Z-Prime Plus explicitly in the abstract sentence. Manufacturers are detailed in Methods/Table 1 per journal practice; to keep the abstract lean and non-promotional, company names are not repeated there.

“Primers were a universal adhesive (Single Bond Universal, 3M) or a primer containing 10-methacryloyloxydecyl dihydrogen phosphate Z-Prime Plus (Bisco), and adhesives were a light-cure orthodontic composite or a dual-cure resin cement.”

***Comment:*** - "Shear bond strength was analyzed with three-way ANOVA." Please detail any post hoc tests that were performed.

***Response:*** Clarified in the abstract:

“One hundred twenty-eight specimens (n=16 per group) were tested. Shear bond strength was analyzed with three-way ANOVA, followed by post-hoc Tukey tests.”

***Comment:*** - What does "fabrication x primer" mean? Do you want to stick to symbol × here?

***Response:*** Replaced “x” with the multiplication symbol **×** throughout and defined once as an interaction term.

***Comment:*** - "Clinically, customized lingual bases fabricated by either additive manufacturing or conventional casting can provide reliable bonding (...)." You have not studied anything clinically. Revise carefully.

***Response:*** Thank you. We have rephrased to avoid clinical inference:

“Within this *in vitro* model, base manufacturing may warrant attention, whereas primer and curing mode may be selected for handling and workflow considerations, with clinical relevance yet to be established.”

***Comment:*** - With your Conclusions, please stick exclusively to your (primary/secondary) aims, and follow your aims' order. Do NOT simply repeat your results here. Do not speculate. Do not provide well-accepted but meaningless phrases. Instead, provide a reasonable and generalizable extension of your outcome.

***Response:*** Thank you for your comment. Conclusions were streamlined to map directly to the stated aims: (1) fabrication modality was the primary determinant of retention; (2) primer type and polymerization mode showed no significant effect on shear bond strength; (3) failure modes predominantly occurred at/near the zirconia–adhesive interface.

“On glazed, sandblasted zirconia, shear bond strength of customized lingual brackets showed a borderline main effect of fabrication method, whereas primer type and adhesive polymerization mode were not statistically significant. Failures were predominantly located at or near the zirconia–adhesive interface. Within this *in vitro* model, base manufacturing may warrant attention, whereas primer and curing mode may be selected for handling and workflow considerations, with clinical relevance yet to be established.”

***Comment:*** Intro
- No numbering of headlines.

**Response:** Done. We removed numeric prefixes from all section headings (e.g., “1. Introduction” → “Introduction”) to match the journal style.

***Comment:*** - Please revise for uniform Journal style. See "in-vitro" (and compare with your Title). No dash, please. Please italicize. Revise thoroughly.

***Response:*** Completed. We standardized all instances to italic *in vitro* (no hyphen) across the title, abstract, main text, figures, and tables.

***Comment:*** Methods
- Again, no numbering, please.

***Response:*** Done. Numeric prefixes were removed from “Materials and methods” and all other headings for consistency.

***Comment:*** - "The calculation assumed a medium effect size of 0.25, an alpha level of 0.05, and a statistical power of 0.80. The required total sample size was 128, with 16 specimens per group and equal allocation across the eight experimental cells." Rationale remains unclear. Please provide details of your sample size calculation.

***Response:*** We have added full details of the a priori power analysis (design, software, inputs, and justification). The text now reads:

“An a priori sample-size calculation was performed with G*Power (version 3.1; Heinrich Heine University Düsseldorf, Düsseldorf, Germany) using F tests (ANOVA: fixed effects, special, main effects and interactions) for a three-way ANOVA (2×2×2 between-subjects) with eight groups and numerator degrees of freedom of 1 for each main effect and interaction. The calculation assumed a medium effect size of 0.25 (the smallest effect of interest, based on prior in-vitro bonding reports), an alpha level of 0.05, and a statistical power of 0.80 [17,18]. The required total sample size was 128, with 16 specimens per group and equal allocation across the eight experimental cells.”

***Comment:*** - Why did you treat "two samples with All-Bond Universal"?

***Response:*** This was a typographical error. No specimens were treated with All-Bond Universal. All instances have been corrected to Single Bond Universal (3M) throughout the manuscript (Abstract, Methods/Materials, tables/figures, and dataset). This correction does not affect group definitions, sample counts, results, or conclusions.

“For qualitative surface characterization, an additional six zirconia specimens and six brackets per bracket type were examined by scanning electron microscopy (SEM) (Quanta 450 FEG, Thermo Fisher Scientific, Hillsboro, OR, USA) at 20 kV, including two left unprimed, two treated with Single Bond Universal, and two treated with Z-Prime Plus.”

***Comment:*** - "(...) ranging from 0 to 3.[18,19]" must read "(...) ranging from 0 to 3 [18,19]." Revise carefully.

***Response:*** Corrected throughout to include a space before bracketed citations. All similar instances have been revised for consistency with journal style.

***Comment:*** - "x5 magnification" must read "5× magnification". Again, use symbol ×.

***Response:*** Standardized to use the multiplication symbol and correct order. This change has been applied consistently in the text, figure captions, and tables.

***Comment:*** Results
- "(...) surface roughness (Ra) measured 0.813 ± 0.145 µm (...)" - meaning would seem unclear. Do you stick to means [SD] here? If yes, please revise for "(...) mean [SD] surface roughness (Ra) measured 0.813 [0.145] µm (...)". Please clarify, and revise thoroughly.

***Response:*** Revised throughout to mean [standard deviation (SD)] format for clarity.

“After sandblasting, the mean [standard deviation (SD)] surface roughness (Ra) was 0.81 [0.15] µm for zirconia, 6.26 [1.33] µm for 3D-printed Co-Cr, and 2.12 [0.43] µm for cast Ni-Cr.”

***Comment:*** - Why do you stick to 3 digits here? Avoid any spurious accuracy, please.

***Response:*** To avoid spurious precision, values are rounded to two decimals; p-values are reported to three decimals (p<0.001 when smaller).

***Comment:*** - Again,. please revise for uniform Journal style. Compare "(P<.001)" (full text) and "(P<0.001)" (Abstract section). Do you see you non-uniform style?

**Response:** We standardized p-value formatting across the manuscript to “P=0.XXX” (exact) and “P<0.001” (when appropriate)

***Comment:*** - Same with "P <0.001", and with "P = 0.017". Revise thoroughly.

**Response:** We standardized p-value formatting across the manuscript to “P=0.XXX” (exact) and “P<0.001” (when appropriate)

***Comment:*** Disc
- "(...) validated for orthodontic components.[20,21]" - see comments given above. Again, revise thoroughly for sound Journal style.

***Response:*** Corrected throughout to include a space before bracketed citations. All similar instances have been revised for consistency with journal style.

***Comment:*** - What about the strengths of your study?

**Response:** We added a short “Strengths” paragraph in Discussion highlighting the factorial design (2×2×2) with balanced allocation and a priori power, standardized zirconia glazing/abrasion, complementary SEM–roughness analyses, blinded ARI scoring, and the use of clinically relevant primers/adhesives and manufacturing routes.

“This study has several strengths. The 2×2×2 factorial design with equal allocation and an a priori power analysis enabled simultaneous estimation of main effects and interactions with balanced precision. Surface preparation and zirconia glazing were standardized, and complementary Ra measurements and SEM micrographs provided mechanistic context to the bond-strength outcomes. Adhesive remnant evaluation was performed with blinded scoring, and the tested materials (MDP primer, universal adhesive, light-cure composite, dual-cure cement) reflect common clinical practice for bonding to glazed zirconia.”

***Comment:*** - There would seem more limitations, please discuss.

**Response:** We expanded the “Limitations” paragraph to cover: (i) in-vitro setting without cyclic fatigue/chewing pH dynamics; (ii) single zirconia system and one abrasion protocol; (iii) brand-specific materials; (iv) unmeasured conversion/film thickness/wettability; (v) use of flat zirconia coupons; (vi) SBS as the sole metric; and (vii) important confounding between fabrication route and alloy, which prevents isolating manufacturing from alloy chemistry.

“Flat zirconia substrates were used rather than anatomic restorations, and tensile bond strength was not evaluated. Furthermore, fabrication route and alloy were not fully orthogonal, so differences attributed to “fabrication” may partly reflect alloy-specific topographic or chemical effects. Future investigations addressing multiple alloys, substrates, surface protocols, and loading modes will be needed to confirm generalizability.”

***Comment:*** - What about any responses to your teaching objectives?

**Response:** We added a brief “Teaching points/Implications for practice” subsection summarizing actionable takeaways for clinicians and lab technicians.

“In practical terms, for bonding customized lingual brackets to glazed zirconia, manufacturing modality was the main driver of retention in this model, albeit a borderline effect on bond strength. Additively manufactured Co–Cr bases showed higher shear bond strength than cast Ni–Cr, particularly with light-cure adhesives, with differences attenuated under dual-cure cements. Primer choice did not meaningfully change mean bond strength; thus, selection can be guided by handling and workflow. Notably, 3D-printed bases were associated with lower ARI levels than cast (especially with MDP primer), which may reduce chairside cleanup of the zirconia surface. Routine airborne-particle abrasion remains essential for zirconia, and curing strategies should account for light attenuation under opaque ceramics.”

***Comment:*** Concl
- Again, with your Conclusions, please stick exclusively to your primary/secondary aims, and follow your aims' order. Do NOT simply repeat your results here. Do not speculate. Do not provide well-accepted but meaningless phrases. Instead, provide a reasonable and generalizable extension of your outcome.

Response: Thank you for your comment. The Conclusions section have been revised accordingly:

“Within an *in vitro* 2×2×2 design on glazed, sandblasted zirconia, shear bond strength showed a borderline main effect of fabrication, with additively manufactured Co–Cr customized bases associated with higher values than cast Ni–Cr counterparts. Primer type and polymerization mode did not show meaningful main effects, and no interactions were detected. Failures occurred predominantly at or near the zirconia–adhesive interface. Given the modest effect size and in-vitro context, clinical implications remain uncertain; at most, base manufacturing method may be considered when other factors are comparable, whereas primer and curing mode can be selected based on handling and workflow until confirmed in clinically representative studies.”

***Comment:*** Refs
- Revise for uniform Journal style.
Several minor and major revisions would seem mandatory with this submitted draft.

***Response:*** Completed. We harmonized citation punctuation, author initials, journal abbreviations, volume/issue/pages, DOI formatting, and bracket spacing to the journal style throughout the reference list.

--------------------------------

**Reviewer #2:** 
***Comment:*** • The points made in the introduction and discussion are fine, but include a few lines explaining how your findings were justified and how they were compared to those of other recent, pertinent studies.

***Response:*** Thank you for the helpful suggestion. We have revised the Introduction and Discussion section accordingly:

“Despite the growing adoption of additive manufacturing for orthodontic appliances, no study has clarified whether the distinct surface topography and alloy microstructure of three-dimensionally (3D) printed brackets influence bonding behavior on zirconia compared with traditional cast counterparts. Establishing these differences is clinically relevant because mismatched surface characteristics can alter micromechanical retention and chemical coupling, ultimately affecting bracket reliability on zirconia restorations.”

“In prior in vitro studies, bonding to zirconia is largely mechanochemically driven, with airborne-particle abrasion being essential [10]. Once an effective primer is applied, both light-cure orthodontic composites and dual-cure resin cements routinely achieve bond strengths of 8–15 MPa [12-14]. Without a primer, light-cure composites generally underperform and often fail after thermocycling, whereas dual-cure and self-adhesive systems containing functional phosphates can partially compensate [10,11,13]. Dedicated MDP primers and one-bottle universal adhesives tend to yield comparable shear bond strength on abraded zirconia. Reported differences are small and inconsistent across studies, with universal agents sometimes showing higher initial wettability but similar aged performance [12-14]. Direct head-to-head data on customized lingual brackets bonded to zirconia are scarce. On enamel, customized lingual brackets typically exhibit low-to-mid shear bond strength (2-6 MPa), yet their enlarged bases yield debonding forces that are equivalent to or can exceed those of stock brackets [15,19]. The lower bond strength in those reports likely reflects that an MDP-containing primer was not applied to the metal bracket base.”

***Comment:*** • The title need revision

***Response:*** Revised to sentence case and italicized *in vitro*, as requested: “Effects of manufacturing modality, primer, and adhesive polymerization on the shear bond strength of customized lingual brackets to glazed zirconia: An *in vitro* study.”

***Comment:*** • You should remove outdated references from your reference list and keep just current ones.

***Response:*** Done. We replaced older/legacy sources with recent (2020–2025) literature where available and removed redundant citations; the reference list now prioritizes contemporary, peer-reviewed evidence.

***Comment:*** • Multiple typing and grammar errors

***Response:*** Corrected. We performed a line-by-line language edit to fix typographical errors, enforce uniform terminology, and standardize units/rounding and statistical notation across text, tables, and figure captions.

--------------------------------

**Reviewer #3:** Dear Editor,
Thank you for the opportunity to review “Effects of manufacturing modality, primer, and adhesive polymerization on the shear bond strength of customized lingual brackets to glazed zirconia: An in vitro study” (PONE-D-25-46749). The topic is timely at the orthodontics–prosthodontics interface, addressing clinical bonding challenges on zirconia with customized lingual brackets. The experimental design is generally sound (2×2×2 factorial; n=16/group; thermocycling), and the paper is clearly written. I provide detailed, constructive comments below to help strengthen methodological transparency, statistical reporting, and clinical interpretation.
***Comment:*** 1. Kindly add the level of significance to the Methods section.

***Response:*** Thank you. We have added the significant level to the Methods section of Abstracts:

“Significance was set at α=0.05.”

***Comment:*** Introduction
6. The introduction outlines zirconia bonding but the knowledge gap is not strongly emphasized. State clearly why comparing 3D-printed vs. cast customized lingual brackets on zirconia is novel and relevant.

***Response:*** We thank the reviewer for this valuable comment. The introduction has been revised to emphasize the specific knowledge gap and novelty of comparing 3D-printed and cast customized lingual brackets bonded to zirconia.

“Despite the growing adoption of additive manufacturing for orthodontic appliances, no study has clarified whether the distinct surface topography and alloy microstructure of three-dimensionally (3D) printed brackets influence bonding behavior on zirconia compared with traditional cast counterparts. Establishing these differences is clinically relevant because mismatched surface characteristics can alter micromechanical retention and chemical coupling, ultimately affecting bracket reliability on zirconia restorations.”

***Comment:*** 7. Hypotheses should be more explicit: state expected effects for fabrication, primer, and polymerization, as well as interactions.

***Response:*** Thank you for the suggestion. We have retained the null hypotheses and added a concise “Pre-specified hypotheses” block that states our directional expectations for fabrication, primer, polymerization, and their interactions, grounded in micromechanical and chemical bonding rationales.

“A priori, it was expected that 3D-printed Co–Cr would yield equal or higher bond strength than cast Ni–Cr owing to rougher post-abrasion bases; that an MDP-containing primer would perform equal to or better than a universal adhesive on zirconia via phosphate–oxide coupling; and that dual-cure would perform equal to or better than light-cure on zirconia because of light attenuation in the substrate.”

***Comment:*** Materials and Methods
9. The phrase “CAD-CAM” is used without expansion at its first mention. The full term “computer-aided design and computer-aided manufacturing (CAD-CAM)” should be provided initially, then the abbreviation may be used thereafter.

***Response:*** Thank you for your comment. The first occurrence of CAD-CAM has been expanded.

***Comment:*** 10. Airborne-particle abrasion: particle size, type, and nozzle angulation are not reported. These are required for reproducibility.

***Response:*** In response, the abrasion parameters have been specified: particle type (aluminum oxide), particle size (110 µm), and nozzle angulation (90°, perpendicular to the surface).

“Then, both the glazed zirconia surfaces and the bracket bases were subjected to airborne-particle abrasion using 110-µm aluminum oxide for 20 sec at 0.3 MPa, delivered perpendicular to the surface (90°) from a 10-mm stand-off distance.”

***Comment:*** 11. «Surface roughness (Ra) was also measured on zirconia substrates, although all specimens underwent the same airborne-particle abrasion treatment. Since zirconia surface preparation was not a variable in the experimental design, the inclusion of Ra values for zirconia appears supplementary rather than hypothesis-driven. The authors should clarify the rationale and intended role of zirconia roughness measurement within the study (e.g., descriptive control vs. analytical variable).»

***Response:*** We retained zirconia Ra measurement as a descriptive quality-control variable to verify consistent substrate preparation and to contextualize failure modes. Zirconia Ra was **not** part of hypothesis testing and is reported descriptively to document process uniformity and the expected roughness asymmetry versus bracket bases. We have clarified this in Methods and Discussions:

“Surface roughness of zirconia was recorded as a process-control measure to verify preparation consistency and to contextualize failure modes.”

“Across groups, the predominance of ARI 0–1 indicates failures closer to the zirconia-adhesive interface. This pattern is expected because abraded zirconia remains smoother than the metal bracket bases, whereas the latter’s higher post-abrasion roughness yields stronger micromechanical interlocking and bracket-side retention.”

***Comment:*** 12. Clarify whether bonding occurred to abraded glaze or to exposed zirconia after sandblasting, since this changes the mechanism of bonding.

***Response:*** Bonding occurred to exposed zirconia, not to an intact glaze. Under the present APA parameters (110-µm Al₂O₃, 0.3 MPa, 20 s, 10 mm, 90°), the thin glaze layer (typically ~20–50 µm) is expected to be largely removed. We have revised the Methods and Results section accordingly:

“Under these parameters, the procedure was intended to deglaze and expose the zirconia substrate, avoiding bonding to the glaze, which adheres weakly to zirconia and is prone to debonding [5,20].”

“For zirconia surfaces, no continuous glassy glaze layer was observed, consistent with exposure of the underlying zirconia.”

***Comment:*** 13. Provide curing light details (brand, model, irradiance, tip size, calibration procedure).

***Response:*** Thank you. We have added curing light details to the Method section:

“Light activation was performed with a Led.F (Woodpecker, Guilin, China) curing unit (tip diameter 8 mm); irradiance at the tip was measured as 1200 mW/cm² with an accompanying radiometer before each specimen. Each margin (mesial, distal, occlusal, and gingival) was light-cured for 20 sec.”

***Comment:*** 14. Adhesive film thickness control is not described; this is important since polymerization mode was tested.

***Response:*** Thank you. Film thickness was standardized by controlled seating force and timed seating before light activation:

“In both cases, the bracket was seated on the zirconia surface using a 3D-printed transfer jig under a constant 10-N load, excess was removed with an explorer.”

***Comment:*** 15. State whether the SBS test followed ISO 29022 or another standard. If not, acknowledge.

***Response:*** The test followed the notched-edge shear (chisel) approach per ISO 29022 where applicable (loading geometry and crosshead speed), with the deviation that specimens were bracket–zirconia assemblies:

“Testing followed ISO 29022:2013 notched-edge shear principles, adapted to bracket–zirconia assemblies [24].”

***Comment:*** 16. Bracket base area: indicate if finishing altered area between printed vs cast bases and how this was handled in normalization.

***Response:*** Thank you. We have revised the Method section to address clarify this issue:

“No support plate was present at the bracket base, and no finishing was performed on the base surfaces; only standardized airborne-particle abrasion was applied. Consequently, the bracket base area was not altered by fabrication method.”

***Comment:*** 17. “The manuscript states that specimens underwent 2000 thermocycles between 5 °C and 55 °C. Please add a reference-supported explanation of how many months or years of clinical service this corresponds to,

***Response:*** Thank you. We now cite commonly used laboratory conversions 2,000 cycles approximate 2.4 months. We also added a limitation acknowleding the limited thermocycling:

“Based on commonly used laboratory conversions, 2,000 cycles approximate 2.4 months of clinical service [22]. This regimen was selected to represent early intraoral aging, the period in which bracket debonding is most likely to occur clinically [23].”

“The thermocycling regimen should be interpreted as an early-aging proxy rather than a simulation of the entire duration of fixed-appliance therapy.”

***Comment:*** 18. Clarify primer application protocol (application time, whether Single Bond Universal was cured or air-thinned only).

***Response:*** Primer protocols are now explicit. Single Bond Universal (SBU) on zirconia was scrubbed for 10 s and air-thinned for 5 s; it was not light-cured prior to seating to avoid creating a pre-polymerized film that could increase thickness and impair bracket fit, consistent with manufacturer guidance for indirect workflows and reviews highlighting film-thickness concerns for indirect bonding:

“The primers were not light cured prior to seating to avoid formation of a pre polymerized film that could increase thickness and impair bracket fit [21].”

***Comment:*** 19. Statistical section: describe checks for normality, variance homogeneity, and outliers.

***Response:*** Thank you for your comment. The Statistical analysis section has been revised accordingly:

“Assumptions for parametric modeling were checked a priori: normality by Shapiro–Wilk tests; variance homogeneity by Levene’s test; and outliers by studentized residuals.”

***Comment:*** 20. Multiple comparison adjustments: manuscript mentions Tukey and Holm — specify which contrasts used which correction.

***Response:*** Thank you for your comment. The Statistical analysis section has been revised accordingly:

“Multiplicity-controlled pairwise comparisons used Tukey’s Honestly Significant Difference (HSD) on model-based estimated marginal means for the simple effects of fabrication×primer, evaluated separately within each polymerization condition. In addition, four simple contrasts compared light-cure vs dual-cure within each fabrication×primer cell using t-tests with Holm adjustment.”

***Comment:*** 21. For ARI scoring, report whether examiners were blinded and whether inter-rater reliability was assessed.

***Response:*** Thank you for your comment. The Statistical analysis section has been revised accordingly:

“Two calibrated examiners, blinded to group allocation, independently scored ARI, and inter-rater reliability was quantified using Cohen’s κ.”

***Comment:*** Results
20. Emphasize that the fabrication effect was marginal (P=0.049) and interactions were not significant.

***Response:*** We now emphasize that the fabrication effect on SBS was *marginal* (P = 0.049) and that all interaction terms were non-significant.

“Regarding shear bond strength, the three-way ANOVA (Table 2) revealed a marginal main effect of fabrication method (F=3.95, P=0.049), with higher values observed for 3D-printed brackets compared with cast brackets. The main effects of primer (F=1.39, P=0.241) and adhesive polymerization (F=1.65, P=0.201) were not significant. None of the interaction effects reached significance, including fabrication×primer (F=0.39, P=0.536), fabrication×adhesive polymerization (F=0.94, P=0.334), primer×adhesive polymerization (F=0.96, P=0.330), and fabrication×primer×adhesive polymerization (F=0.02, P=0.876).”

***Comment:*** 21. For ARI: describe blinding/reliability or note as limitation.

***Response:*** Thank you for your comment. Reliability has been added to Results:

“Inter-rater agreement for ARI scoring was perfect between the two examiners (Cohen’s κ=1.00).”

***Comment:*** 22. State explicitly that all groups (≈9–10 MPa) exceeded the clinical threshold with a citation.

***Response:*** Results is preferred to be concise and largely descriptive without citation, so the comparison of bond strengths with clinical threshold was mentioned in the Discussion section with citations.

***Comment:*** Discussion
25. SEM images should be described as qualitative only, not quantitative proof of film thickness.

***Response:*** We agree and now state explicitly that the SEM micrographs are qualitative illustrations of morphology only; no film-thickness inferences are made, and contrast is interpreted cautiously given coating and imaging effects:

“In SEM images, the nearly dry appearance of the MDP primer after air-drying is consistent with its volatile and unfilled formulation. Because the specimens were coated under vacuum before imaging, residual solvents would have been further removed, potentially accentuating the dry appearance. Consequently, the dark primer remnants are mainly restricted to micro-pits, with more of the ceramic substrate visible. In contrast, the universal adhesive’s water-containing, hydrophilic, and filler-reinforced matrix promotes surface wetting and yields the appearance of a more continuous post-drying film (uniform dark layer) on metallic substrates.”

***Comment:*** 26. Since polymerization mode was non-significant, suggest that future work measure degree of conversion beneath zirconia (e.g., FTIR).

***Response:*** Thank you. The above suggestion has been added:

Future works should directly assess degree of conversion beneath opaque substrates using Fourier-transform infrared spectroscopy (FTIR) to verify and generalize the present findings.

***Comment:*** 27. Limitations should be expanded: one zirconia/glaze, no mechanical fatigue, no conversion data, no adhesive thickness control, potential variability in base area.

***Response:*** Thank you, the limitations have been expanded:

“It was conducted in an *in vitro* setting with static shear loading after 2000 thermocycles and did not include cyclic fatigue, chewing simulation, and pH fluctuations. The thermocycling regimen should be interpreted as an early-aging proxy rather than a simulation of the entire duration of fixed-appliance therapy. A single zirconia and a single airborne-particle abrasion protocol were used, which may limit generalizability to other zirconia systems, glaze conditions, and surface treatments. The materials tested were restricted to one MDP primer, one universal adhesive, one light-cure orthodontic composite, and one dual-cure resin cement, so findings may not extend to other brands or newer chemistries. Potential variability in bracket base area and the lack of direct quantification of the effective bonded area could influence the calculated bond strength. Degree of conversion beneath opaque substrates, adhesive film thickness, and wettability/contact angle were not measured. Flat zirconia substrates were used rather than anatomic restorations, and tensile bond strength was not evaluated. Furthermore, fabrication route and alloy were not fully orthogonal, so differences attributed to “fabrication” may partly reflect alloy-specific topographic or chemical effects.”

***Comment:*** 28. Tone down the conclusion: the only significant effect was fabrication, marginally. Avoid overstating clinical impact.

“Within an *in vitro* 2×2×2 design on glazed, sandblasted zirconia, shear bond strength showed a borderline main effect of fabrication, with additively manufactured Co–Cr customized bases associated with higher values than cast Ni–Cr counterparts. Primer type and polymerization mode did not show meaningful main effects, and no interactions were detected. Failures occurred predominantly at or near the zirconia–adhesive interface. Given the modest effect size and in-vitro context, clinical implications remain uncertain; at most, base manufacturing method may be considered when other factors are comparable, whereas primer and curing mode can be selected based on handling and workflow until confirmed in clinically representative studies.”

***Comment:*** 29. Add a short clinical implications paragraph: how should orthodontists weigh printed vs cast bases, considering both bond strength and ARI distribution.

***Response:*** Thank you for your comment. A short clinical implications paragraph has been added:

“In practical terms, for bonding customized lingual brackets to glazed zirconia, manufacturing modality was the main driver of retention in this model, albeit a borderline effect on bond strength. Additively manufactured Co–Cr bases showed higher shear bond strength than cast Ni–Cr, particularly with light-cure adhesives, with differences attenuated under dual-cure cements. Primer choice did not meaningfully change mean bond strength; thus, selection can be guided by handling and workflow. Notably, 3D-printed bases were associated with lower ARI levels than cast (especially with MDP primer), which may reduce chairside cleanup of the zirconia surface. Routine airborne-particle abrasion remains essential for zirconia, and curing strategies should account for light attenuation under opaque ceramics.”

***Comment:*** Figures & Tables
30. Figure legends should be fully self-contained (define abbreviations, state what the figure shows).

***Response:*** Thank you. We have revised all figure legends accordingly:

**Fig. 1.** Study workflow outlining specimen preparation. Sandblasted zirconia crowns were bonded with customized lingual bracket bases manufactured either by 3D-printing or by casting. Two primers were evaluated: an 10-methacryloyloxydecyl dihydrogen phosphate (MDP) primer and a universal primer. Two polymerization modes were used: a light-cure orthodontic composite and a dual-cure resin cement. After bonding, specimens underwent thermocycling and were tested for shear bond strength.

**Fig. 2.** Representative scanning electron micrographs (1000×). (A–C) Zirconia surfaces after sandblasting, with 10-methacryloyloxydecyl dihydrogen phosphate (MDP) primer, and universal primer. (D–F) 3D-printed bracket bases after sandblasting, with MDP primer, and universal primer. (G–I) Cast bracket bases after sandblasting, with MDP primer, and universal primer.

**Fig. 4.** Representative images of Adhesive Remnant Index (ARI) scores. A, ARI 0: no adhesive remaining on the zirconia surface. B, ARI 1: less than 50% of the adhesive remaining on the zirconia surface. C, ARI 2: more than 50% of the adhesive remaining on the zirconia surface. D, ARI 3: all adhesive remaining on the zirconia surface.

***Comment:*** 31. Reduce redundancy between figures and tables — e.g., avoid duplicating the same numerical results.

***Response:*** Thank you for the helpful suggestion. We agree that duplication should be minimized. We have retained both Fig. 3 and Table 3 because they serve distinct purposes: the box plots transparently show the distributions, spread, and outliers in each cell (useful for assessing assumptions and practical variability), whereas the table provides the exact numerical estimates which are not conveyed by the figure and are important for precise interpretation and future meta-analyses. We hope this balance preserves clarity while avoiding unnecessary repetition.

***Comment:*** References

33. Update with more recent references (2019–2024) on zirconia bonding and digital orthodontics.

***Response:*** Done. We replaced older/legacy sources with recent (2020–2025) literature where available and removed redundant citations; the reference list now prioritizes contemporary, peer-reviewed evidence.

***Comment:*** 34. Add a citation for the clinical threshold of SBS at the first mention.

***Response:*** The citation has been inserted in the revised manuscript.
